# The impact of neighborhood deprivation on mental health and quality of life in children and adolescents during the COVID-19 pandemic: Findings from the COPSY Hamburg study

**DOI:** 10.1371/journal.pone.0313652

**Published:** 2024-11-20

**Authors:** Lydia Yao Li, Ann-Kathrin Napp, Adekunle Adedeji, Michael Erhart, Anne Kaman, Maren Boecker, Tanja Kloster, Anne Caroline Krefis, Franziska Reiß, Ulrike Ravens-Sieberer

**Affiliations:** 1 Department of Child and Adolescent Psychiatry, Psychotherapy and Psychosomatics, University Medical Center Hamburg-Eppendorf, Hamburg, Germany; 2 Department of Health Science, Hamburg University of Applied Science, Hamburg, Germany; 3 Bremen International Graduate School of Social Science, Constructor University, Bremen, Germany; 4 Department of Public Health, Alice Salomon University of Applied Sciences, Berlin, Germany; 5 Department of Psychology and Education, Apollon University of Applied Sciences, Bremen, Germany; 6 Department of Child and Adolescent Psychiatry, Child Neuropsychology Section, University Hospital RWTH Aachen, Aachen, Germany; 7 Institute of Medical Psychology and Medical Sociology, University Hospital of RWTH Aachen University, Aachen, Germany; 8 Department for Health, Hamburg Authority for Work, Health, Social Affairs, Family and Integration, Hamburg, Germany; Tehran University of Medical Sciences, ISLAMIC REPUBLIC OF IRAN

## Abstract

**Introduction:**

Socioeconomic inequalities have been associated with poorer mental health outcomes in children and adolescents during the COVID-19 pandemic. Despite numerous studies on individual risk factors, the impact of societal environment, such as neighborhood characteristics, on changes in mental health has rarely been investigated. This study investigates the effect of neighborhood deprivation on mental health problems and health-related quality of life (HRQoL) in children and adolescents during the COVID-19 pandemic in Hamburg, Germany.

**Methods:**

Data were derived from the prospective German COPSY Hamburg study. Children and adolescents aged between 11–20 years and their parents participated in the study, which took place in summer 2020 (T1) and summer 2022 (T2). Neighborhood deprivation was assessed by a neighborhood status index. Mental health problems and HRQoL were assessed using internationally validated and established instruments. The prevalence of mental health problems and impaired HRQoL was reported. Analysis of covariance was conducted to examine the effect of neighborhood deprivation of the districts in Hamburg on the (changes in) mental health problems and HRQoL while controlling for social individual-level indicators.

**Results:**

The total sample included in the statistical analysis consisted of *N* = 2,645 families. Children and adolescents living in more deprived areas had higher levels of general mental health problems and depressive symptoms during the COVID-19 pandemic. However, differences in neighborhood deprivation did not relate to the HRQoL and the averaged changes in children and adolescents’ mental health problems and HRQoL from summer 2020 to summer 2022.

**Discussion:**

Neighborhood deprivation is associated with impaired mental health in youth during the COVID-19 pandemic. Children and adolescents’ mental health and overall well-being should be addressed by health promotion measures to create a health-promoting living environment, including diverse neighborhoods. Future research should focus on uniform assessment methods and addressing additional neighborhood factors.

## Introduction

The detrimental impact of the COVID-19 pandemic on mental health and health-related quality of life (HRQoL) of children, adolescents, and families has been well documented. Although pandemic-related restrictions have eased since spring 2022 in many regions of Germany [1,2], research has shown long-lasting psychosocial consequences beyond the initial period, indicating a notable increase in symptoms of depression, anxiety, and heightened stress among youths [3,4].

Social isolation resulting from prolonged lockdowns and school closures has deprived young individuals of crucial social interactions, leading to feelings of loneliness and a lack of emotional support [3,5,6]. The pandemic has accentuated social inequality issues, disproportionately affecting vulnerable communities and amplifying existing disparities in education, financial resources, healthcare, and COVID-19-related mortality [7–10]. However, there is evidence showing no increase in, or even a reduction in, inequality regarding mental health problems during the pandemic [11,12]. Nevertheless, children from low-income families showed higher levels of mental health problems than children from higher-income families in pre-pandemic and pandemic studies [3,13]. Economic hardships experienced by families due to job losses or financial instability can lead to increased (parental) stress and emotional burdens, further affecting the mental health of young individuals [14]. Similarly, socioeconomic inequalities have been associated with poorer mental health outcomes during the COVID-19 pandemic in numerous other international longitudinal studies [15–19] and reviews [3,20]. A socioeconomically disadvantaged group of children and adolescents has also been identified by the nationwide COvid-19 and PSYchological Health (COPSY) study in Germany, showing worse mental health outcomes [21], which has been replicated in the first survey time point (T1) of the regional COPSY Hamburg study [22].

Connected with some of the individual-level social determinants mentioned above, neighborhood conditions have emerged as significant determinants of mental health and overall well-being, shaping the experiences of individuals and communities in specific geographical contexts [23–25]. Neighborhood socioeconomic measures can be used to identify deprived areas, allowing targeted public health efforts. There are several measures for area deprivation on a transnational [26], nationwide [11,27,28], or local/urban level [12,29], which are based on various indicators. However, it should be noted that neighborhood-level measures, such as area deprivation, cannot replace individual-level data. Given that both individual and neighborhood socioeconomic measures independently impact health and well-being [30], research on both levels is highly warranted.

The association between area deprivation and mental health is shown in several studies. For example, a systematic review found strong evidence for increased suicidal behavior in more deprived areas in Europe [31]. Another review focusing on young people reported negative associations between growing up in a deprived neighborhood and mental health as well as well-being in the majority of reviewed studies [32].

The impact of neighborhood deprivation on the mental health and HRQoL of children and adolescents during the COVID-19 pandemic has become an area of growing concern and research. A large-scale UK study by Miall and colleagues [11] concluded that inequalities in young children’s mental health related to area deprivation persisted during the pandemic, indicating children from deprived areas had poorer mental health prior to and during the pandemic. However, a decrease in area deprivation inequality was observed when comparing children from the most affluent quintile of areas with those from all other areas [11]. Similar findings were made in the Born-in Bradford-cohort study, which focused on families with 8- to 12-year-olds living in the city of Bradford, UK [12]. Children’s mental health problems increased in all areas during the pandemic. However, findings differed between ethnic groups [12].

These research findings underscore the significance of understanding and addressing individual- and neighborhood-level factors in shaping young individuals’ mental health and well-being. Nonetheless, there is a need for prospective cohort and trend studies that can assess the long-term effects of neighborhood deprivation on children and adolescents in different social and geographic contexts while controlling for individual-level socioeconomic status (SES). Understanding how these factors interact with the pandemic situation over time can provide a comprehensive understanding of their lasting impact on mental health outcomes. Further, identifying the underlying pathways can inform targeted interventions and policies to create supportive environments that promote resilience and well-being during and after this challenging time. As the COVID-19 pandemic and other interconnected crises continue to impact the lives of children and adolescents, addressing these research gaps is essential for developing evidence-based strategies that prioritize their mental health and overall quality of life.

To the best of our knowledge, no previous published studies have explored the relationship between neighborhood deprivation and young people’s mental health during the COVID-19 pandemic in Germany. Therefore, the current study aims to investigate the effect of neighborhood deprivation on mental health problems and HRQoL in children and adolescents in Hamburg, Germany, after two years of living under varying pandemic conditions. The following research questions were addressed:

1. How is the level of neighborhood deprivation associated with mental health problems and HRQoL in children and adolescents during the COVID-19 pandemic while controlling for individual factors?
2. Do differences in neighborhood deprivation in Hamburg relate to the average changes in children and adolescents’ mental health problems and HRQoL from summer 2020 to summer 2022?

## Materials and methods

### Participants & procedure

The prospective COPSY Hamburg cohort study is a combined online survey for children and adolescents and their parents with a permanent residence in the *Free and Hanseatic City of Hamburg*, Germany. Hamburg is the second-largest city in Germany, with more than 1.9 million inhabitants [33]. The study design of the COPSY Hamburg study was developed in accordance with the nationwide German COPSY study [21].

The first survey (T1) was conducted from June 12^th^ to July 31^st^, 2020, after a Hamburg-wide lockdown with school closures and restrictions on daily life. Overall, *N* = 1,642 families (*n* = 1,484 children and adolescents aged 11 to 17 years and *n* = 1,195 parents) provided data in summer 2020. Detailed information about the recruitment process and weighting procedure is provided in a study by Kaman and colleagues [22].

**Fig 1:**
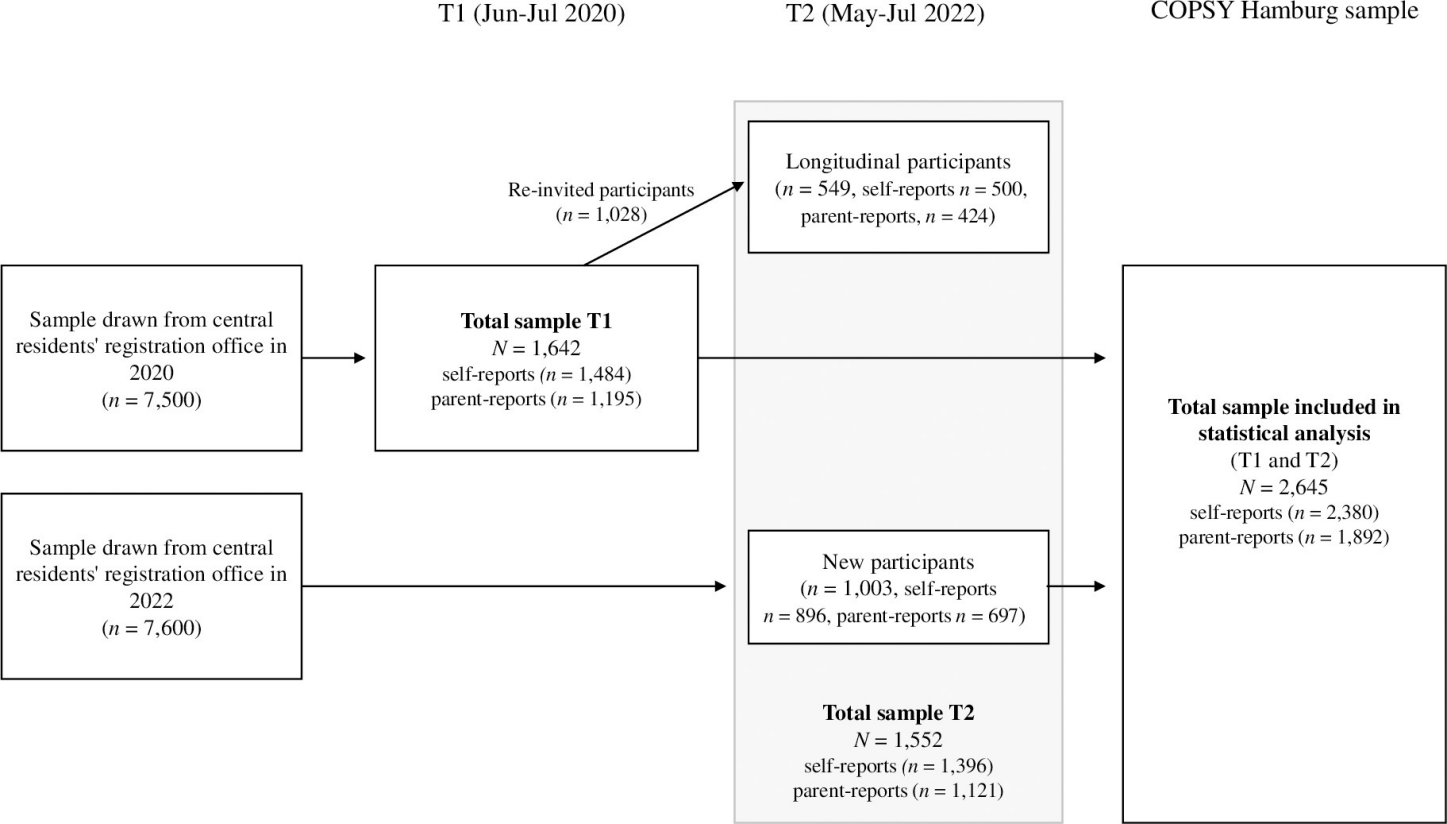
Flow chart of participants of the COPSY Hamburg study from 2020 (T1) and 2022 (T2).

The second survey (T2) took place between May 17^th^ and July 7^th^, 2022, during a period of low infection rates and relaxed restrictions. For this second time point, *N* = 1,028 participants from T1, who had given their consent to be contacted again, were invited to participate. To maintain the representativeness of the COPSY Hamburg study and compensate for the loss of follow-up participants, an additional new sample of *N* = 7,600 children and adolescents was drawn from the central residents’ registration office and invited to participate. Families from disadvantaged neighborhoods were oversampled by randomly sampling from pre-defined adjacent districts or neighborhoods in clusters. Overall, *N* = 1,552 families (*n* = 1,396 children and adolescents aged 11 to 17 years and *n* = 1,121 parents) provided data in the second survey, out of which *n* = 549 families were part of the first survey. The data were weighted to correspond to the sociodemographic characteristics of parents with children between 11 and 18 years in Hamburg (according to the 2020/2021 Microcensus and Population forecast). The total sample included 2,645 families participating in the COPSY Hamburg study in T1 and T2 (Fig 1).

The study was approved by the Local Psychological Ethics Committee at the Center for Psychosocial Medicine (LPEK; T1: LPEK-0151, T2: LPEK-0463) of the University Medical Center Hamburg-Eppendorf (UKE). Written informed consent to participate in this study was provided by the participants’ legal guardian/next of kin.

### Measures

#### Sociodemographic

Information about children and adolescents age and gender was retrieved from the residents’ registration office. Parents self-reported their age and gender. Parental education levels were provided by parents or children if parent reports were unavailable and were classified as *low*, *medium*, or *high* according to the Comparative Analyses of Social Mobility in Industrial Nations (CASMIN). A low education level comprises families who do not have at least a secondary school leaving certificate. The medium education category includes those families who do not have at least a degree from a university of applied sciences. If parents have such a qualification, the family was assigned to the high group [34].

#### Neighborhood deprivation

For operationalizing neighborhood deprivation, a status index according to the *Integrated Neighborhood Development* (Rahmenprogramm Integrierte Stadtteilentwicklung; RISE) Social monitoring was used to assign each participating household to one of four status groups based on their address: *very low*, *low*, *medium*, and *high*. This neighborhood status index has been calculated since 2010 yearly on behalf of the *Ministry for Urban Development and Housing (BSW)* of the Free and Hanseatic City of Hamburg for 853 statistical areas (as of 31.12.2021) with a minimum of 300 inhabitants. Even though the index has not yet been used in similar scientific articles, Pohlan and Strote [35] recommend linking social status data with health data to integrate health aspects into urban development. The index comprises seven indicators, including (1) the proportions of children and adolescents with a migrant background, (2) children with single parents, (3) recipients of basic welfare benefits, (4) unemployed rate, (5) inhabitants under 15 years in need of welfare benefits, (6) recipients of minimum welfare benefits in old age, and (7) proportion of school leavers without qualification or with basic/middle school certificate. The index describes the deviation of each statistical area from the average of all statistical areas in Hamburg. A deviation > –1 SD indicates a *high* neighborhood status index, ± 1 SD a *medium* index, > 1 SD to 1.5 SD a *low* index and > 1.5 SD a *very low* index [35–37]. Participants in COPSY Hamburg T1 were assigned the neighborhood status index from 2020, while those in T2 were assigned the index from 2022. For participants who participated in both surveys, T1 and T2, the neighborhood status index from 2022 was used. No neighborhood status index was available for *n* = 1 household due to insufficient residents in this statistical area. For this household, the neighborhood status index of the neighboring statistical area was used.

#### Mental health

Mental health problems were assessed using the four problem scales of the Strengths and Difficulties Questionnaire [38] via parent report, which has been found to be a reliable and valid screening tool for use in Germany [39]. Sum scores from the conduct and hyperactivity subscales were added up to obtain a score for *externalizing difficulties*, while sum scores from the emotional and peer problems subscales were added up to obtain a score for *internalizing difficulties*. The values for internalizing and externalizing difficulties range between 0 and 20. Higher scores indicate higher levels of mental health problems. Using established German cut-offs [40], children and adolescents were categorized into those with (*abnormal/borderline*) and without (*normal*) mental health problems.

Depressive symptoms were self-reported by children and adolescents using seven items of the German version of the Center for Epidemiological Studies Depression Scale (CES-DC), which have been previously used in the BEhaviour and WeLLbeing of Children and Adolescents in Germany (BELLA) study [41] and the national COPSY study. A sum score (ranging from 7 to 28) was calculated across the seven items, with higher values indicating higher levels of depressive symptoms.

Anxiety symptoms were self-reported by children and adolescents using the nine-item general anxiety subscale of the Screen for Child Anxiety Related Disorders (SCARED). The German version of the SCARED showed good internal consistency in a validation study in a sample of German school children [42,43]. Sum scores range between 0 and 18, with higher values indicating higher levels of anxiety.

Participants were categorized into those with (cut-off CES-DC > = 15; SCARED > = 9) and without depressive and anxiety symptoms using validated cut-offs applied to participants [42,44].

Parental depressive symptoms were assessed using the validated German translation of the eight-item version of the Patient Health Questionnaire (PHQ-8) [45–47] via parent report. A *severity score* between 0 and 24 was calculated, and parents were assigned to two groups with (scores 10–24) and without (scores 0–9) depressive symptoms.

#### Health-related quality of life

Subjectively perceived HRQoL of children and adolescents was assessed using the German version of the KIDSCREEN-10 Index [48]. Children and adolescents self-reported on physical, mental and social aspects of HRQoL on a 5-point-Likert scale. Higher scores indicate higher HRQoL. The KIDSCREEN-10 is a widely used and internationally validated instrument for the assessment of HRQoL and has shown good psychometric properties [49]. T-values were calculated on the basis of Rasch person parameters from a European norm sample [48]. Based on pre-pandemic reference data from the German BELLA study, participants were assigned to three groups with low, medium and high HRQoL. Medium HRQoL was defined as M_BELLA_ +/- 1 SD_BELLA._ [50].

### Statistical analysis

The data of both time points were analyzed with descriptive statistical methods. The mean, standard deviation and 95% confidence intervals were calculated for internalizing and externalizing difficulties, depressive and anxiety symptoms, and health-related quality of life. Cases with missing values in a single variable were excluded for this particular analysis but included in other analyses if possible. Due to the relatively low percentage of respondents participating in both measurement time points, each time point (T1 and T2) was treated as a separate sample in the statistical analysis. Thereby the analysis omits the usage of statistics for repeated measures which have a higher statistical power. On the other hand, the inclusion of the same respondents and analyzing them as separate samples lead to an underestimation of the overall variation and thus a higher chance for significant results. Both effects might cancel out each other.

Bivariate point biserial or pearson correlations between all mental health outcomes and predictors (age, gender, parental education, parental depressive symptoms, neighborhood status index and sample group (T1/T2)) were computed.

The effect of time on each outcome was examined with analysis of covariance (ANCOVA) according to the generalized linear model (GLM) with T1 vs. T2 group as main-effect and age, gender, parental education (CASMIN), parental depression (PHQ-8) as well as neighborhood status index as covariates. The age*gender and neighborhood status index*sample groups (T1/T2) interactions were included as covariates.

A power analyses was performed with G-Power 3.1.9.7. [51] for a GLM with 2 sample groups, 9 covariates (age, gender, low parental education, medium parental education, parental depressive symptoms, very low neighborhood status index, low neighborhood status index, medium neighborhood status index, time), 4 interactions (age*female gender; time*very low neighborhood status index; time*low neighborhood status index; time*medium neighborhood status index). To detect a medium effect size (‘f’ = 0.25) with a Power of 0.95 and an alpha error of 0.05 a total sample size (both time points) of 436 is warranted. Thus, the actual sample size was 2–3 times higher in each model.

All other statistical analyses were performed with IBM SPSS 27.

## Results

### Study characteristics

A total of *N* = 1,642 (T1) and *N* = 1,552 (T2) families participated in the COPSY surveys in summer 2020 and 2022. Sample description is provided in Table 1. Overall, the participating children, adolescents and parents at the first and second time points were similar in terms of age and gender. This includes families with children and adolescents between 11 and 20 years (T1: M_age_ = 13.91 years (50.30% female), T2: M_age_ = 14.51 years (48.71% female)). Of these, *n* = 1,484 (90.38%) children and adolescents provided self-reported data at T1 and *n* = 1,396 (89.95%) children and adolescents provided self-reported data at T2. Parent reported data are available for *n* = 1,195 (72.78%) parents at T1 and *n* = 1,121 (72.23%) parents at T2. Participating parents were mostly female (T1: 73.72%, T2: 76.77%) and on average 47 to 48 years old. The majority of families had a medium or high level of education and lived in neighborhoods with a medium neighborhood status index (T1: 63.52%, T2: 54.45%). At the second time point, one in three families lived in more deprived neighborhoods (very low or low neighborhood status index).

**Table 1:** Sample description of participants in the COPSY Hamburg study.

	COPSY Hamburg T1 (2020)			COPSY Hamburg T2 (2022)		
	*n*	(%)	*M (SD)*	*n*	(%)	*M (SD)*
**Participating families**	1642	100%		1552		100%
**Children and adolescents**						
Available self-reports	1484	90.38%		1396	89.95%	
Age^1^			13.91 (1.97)			14.51 (2.36)
Gender^1,2^						
female	826	50.30%		756	48.71%	
male	816	49.70%		796	51.29%	
**Parents**						
Available parent-reports	1195	72.78%		1121	72.23%	
Age^3.4^			47.84 (5.97)			46.83 (6.59)
Gender^3,5^						
female	881	73.72%		856	76.77%	
male	306	25.61%		258	23.14%	
diverse^6^				1	0.09%	
**Parental education** ^7^						
low	24	2.13%		132	9.35%	
medium	285	25.33%		616	43.63%	
high	816	72.53%		664	47.03%	
**Neighborhood status index** ^8^						
very low	87	5.30%		275	17.72%	
low	79	4.81%		241	15.53%	
medium	1043	63.52%		845	54.45%	
high	433	26.37%		191	12.31%	

*Notes*: Unweighted data

^1^ according to residents’ registration office

^2^ n = 7 (T1) / n = 26 (T2) children and adolescents self-reported their gender as diverse

^3^ parent-reported

^4^ missing data for n = 2 (T1) / n = 8 (T2)

^5^ missing data for n = 8 (T1) / n = 6 (T2)

^6^ answer option diverse only available in T2

^7^ according to CASMIN, missing data for n = 70 (T1) / n = 140 (T2)

^8^ Neighborhood status index according to RISE.

### Mental health and health-related quality of life

The results of the descriptive analysis for mental health outcomes and health-related quality of life in the COPSY Hamburg study in 2020 (T1) and 2022 (T2) are presented in Table 2.

**Table 2:** Descriptives for mental health outcomes and HRQoL in children and adolescents from the COPSY Hamburg study in 2020 and 2022.

		N	Mean (SE)	95% CI	Prevalence/ Proportion impaired
T1 (2020)^3^^,^^4^	HRQoL (KIDSCREEN)^1^	1457	47.84 (0.22)	[47.42; 48.27]	27.25
	Internalizing symptoms (SDQ)^2^	1157	3.07 (0.09)	[2.9; 3.24]	14.15
	Externalizing symptoms (SDQ)^2^	1159	3.95 (0.09)	[3.77; 4.14]	8.97
	Depressive symptoms (CES-DC)^1^	1454	11.9 (0.1)	[11.7; 12.1]	21.94
	Anxiety symptoms (SCARED)^2^	1444	6.1 (0.12)	[5.87; 6.33]	28.88
T2 (2022)^4^	HRQoL (KIDSCREEN)^1^	1386	46.52 (0.24)	[46.05; 46.98]	30.8
	Internalizing symptoms (SDQ)^2^	1115	3.95 (0.11)	[3.73; 4.16]	20.81
	Externalizing symptoms (SDQ)^2^	1115	4.11 (0.1)	[3.92; 4.31]	9.69
	Depressive symptoms (CES-DC)^1^	1373	12.46 (0.11)	[12.25; 12.68]	27.9
	Anxiety symptoms (SCARED)^2^	1371	7.66 (0.14)	[7.39; 7.92]	39.82

*Notes*: ^1^ Self-reported by children and adolescents

^2^ Parent-reported

^3^ Differences from the prevalence rates reported by Kaman et al. (2021) result from differing subsamples

^4^ The samples from T1 and T2 were weighted according to different criteria described in the Materials and methods section. HRQoL: Health-related quality of life.

Findings indicate that prevalence rates for internalizing or externalizing mental health problems, depressive or anxiety symptoms as well as low health-related quality of life were slightly higher in summer 2022 compared to summer 2020. In detail, between 8.97% and 28.88% of the respondents met the particular criteria for mental health problems or low health-related quality of life in 2020 (T1). The particular proportions ranged from 9.69% to 39.82% in 2022 (T2). The parent reported prevalence of internalizing symptoms was higher than prevalence of external symptoms at both time points: Overall, 20.18% children and adolescents showed exhibiting internalizing symptoms including such as emotional problems at T2 compared to 14.15% at T1 (p < .001). Further, 9.69% of children and adolescents showed externalizing symptoms at T2 compared to 8.97% at T1 (p < .001). The strongest increase was shown in anxiety symptoms. At time point 2, 39.82% of the participating children and adolescents reported symptoms of generalized anxiety, compared to 28.88% at T1 (p < .001). Depressive symptoms were lower at T1 (21.94%) compared to 27.9% at T2 (p < .001). The proportion of respondents with low health-related quality of life was slightly higher in 2022 (30.8%) compared to from 27.25% in 2020 (p = .037).

### Neighborhood deprivation, mental health and health-related quality of life

Living in an area with a higher level of neighborhood deprivation (neighborhood status index *very low/low*) was significantly associated with on average higher values in internalizing and externalizing mental health problems, higher values in depressive and anxiety symptoms and lower health-related quality of life. Results of the bivariate correlations for mental health outcomes and predictors are presented in S1 Table.

To test differences between the time points and examine the association with potential confounders and risk factors, multivariate generalized linear model (GLM) analyses were conducted for each outcome. Results of the GLM for mental health problems and health-related quality of life in children and adolescents are presented in Table 3.

**Table 3:** GLM results for mental health problems and health-related quality of life in children and adolescents.

	HRQoL (KIDSCREEN-10)	Internalizing symptoms (SDQ)	Externalizing symptoms (SDQ)	Depressive symptoms (CES-DC)	Anxiety symptoms (SCARED)
Parameters	B (Sig.)	B (Sig.)	B (Sig.)	B (Sig.)	B (Sig.)
Constant	**64.12** **(< 0.001)**	**2.64** **(< 0.001)**	**6.55** **(< 0.001)**	**3.56** **(< 0.001)**	**-3.88** **(0.045)**
Age	**-1.24** **(< 0.001)**	**0.10** **(0.047)**	**-0.17** **(< 0.001)**	**0.63** **(< 0.001)**	**0.84** **(< 0.001)**
Female gender	-4.72	**2.76** **(0.004)**	**2.73** **(0.003)**	**3.47** **(0.005)**	2.55
Age*Female gender	**0.51** **(0.006)**	**-0.22** **(0.001)**	-0.11	**-0.33** **(< 0.001)**	**-0.38** **(< 0.001)**
Parental education^1^ ^2^					
Low education	-0.18	-0.49	**-1.03** **(0.001)**	-0.39	0.36
Medium education	0.52	**-0.40** **(0.014)**	**-0.50** **(0.001)**	-0.30	**-0.49** **(0.040)**
Parental depressive symptoms (PHQ-8)	**-3.63** **(< 0.001)**	**2.87** **(< 0.001)**	**1.88** **(< 0.001)**	**1.78** **(< 0.001)**	**1.23** **(< 0.001)**
Neighborhood status index^3^					
Very low	-1.25	**1.09** **(0.006)**	**1.16** **(0.002)**	0.89	0.70
Low	-1.18	0.51	0.56	**1.27** **(0.013)**	0.93
Medium	-0.25	0.05	0.06	0.47	0.34
Participation T1⁴	0.53	-0.57	-0.03	0.21	**-0.97** **(0.047)**
Interaction time*Neigh-borhood status index					
T1*very low⁵	-0.16	-0.87	-0.18	-0.16	-0.51
T1*low⁶	0.53	0.12	-0.39	-1.20	-0.46
T1*medium⁷	0.04	0.09	0.17	-0.27	0.04
R-Square	0.118	0.140	0.137	0.124	0.212

*Notes*: ^1^ assessed by CASMIN; Reference categories

^2^ high education

^3^ high status index

^4^ T2

^5^ T2*very low

^6^ T2*low

^7^ T2*medium.

When comparing the most deprived neighborhoods (neighborhood status index *very low*) with the most affluent areas (neighborhood status index *high*), internalizing and externalizing problems were increased (internal: Δ-mean = 1.09, *p* = 0.006; external: Δ-mean = 1.16, *p* = 0.002). Further, living in an area with a *low* neighborhood status index was associated with higher depressive symptoms (CES-DC) (Δ-mean = 1.27, *p* = 0.013). No significant associations were found for level of neighborhood deprivation and anxiety symptoms as well as health-related quality of life in children and adolescents in the multivariate generalized linear model.

None of the interactions between level of neighborhood deprivation and time was significant, meaning that differences in neighborhood deprivation in Hamburg did not relate to the averaged changes in children and adolescents’ mental health problems and health-related quality of life between the two time points.

A statistically significant effect of time (participation in COPSY Hamburg T1 or T2) was only found for anxiety symptoms with a lower mean score on the SCARED for participants of T1 (Δ-mean = 0.97, *p* = 0.047; Table 3).

Older age was associated with lower HRQol (Δ-mean = -1.24, *p*<0.001) and higher mean values in internalizing problems (Δ-mean = 0.097, *p*<0.047), depressive symptoms (Δ-mean = 0.625, *p*<0.001) and anxiety symptoms (Δ-mean = 0.843, *p*<0.001). However, older participants exhibited slightly lower scores on the externalizing problem subscale of the SDQ (Δ-mean = -0.166, *p*<0.001).

Female gender was associated with higher values in both, internalizing (Δ-mean = 2.76, *p* = 0.004) and externalizing (Δ-mean = 2.73, *p* = 0.003) symptoms as well as more pronounced depressive symptoms (Δ-mean = 3.47, *p* = 0.005). In females, higher age was associated with higher HRQoL (Δ-mean = 0.51, *p* = 0.006) and lower mental health problems in all other outcomes except for externalizing symptoms.

Parental education had a significant relation with internalizing and externalizing mental health problems (SDQ) and anxiety symptoms. More specifically, a low or medium level of parental education was significantly associated with lower mean scores on mental health (internalizing problems: medium vs. high Δ-mean = -0.40, *p* = 0.014; externalizing problems: low vs. high Δ-mean = -1.03, *p* = 0.001; medium vs. high Δ-mean = -0.50, *p* = 0.001) and anxiety symptoms (medium vs. high Δ-mean = -0.49, *p* = 0.040).

Parental depressive symptoms were associated with reduced mean HRQoL (Δ-mean = -3.63, *p*<0.001) and higher mean scores for internalizing and externalizing symptoms, depressive and anxiety symptoms (Δ-mean = 1.23 to 2.87, *p*<0.001).

Overall, the GLM Models explained between 11.8% and 21.2% in children and adolescents’ mental health outcomes and health-related quality of life.

## Discussion

This is the first study combining children and adolescents’ mental health with macro-data about neighborhood deprivation while controlling for individual risk factors in a German metropole during the COVID-19 pandemic.

With reference to our first research question, children and adolescents living in neighborhoods with the highest level of neighborhood deprivation in Hamburg have worse mental health and health-related quality of life compared to those living the most affluent areas of Hamburg.

To give an example, living in an area with the lowest neighborhood status index was associated with higher values in internalizing symptoms of children and adolescents. These results are in line with other studies [11,12,52], which reported that neighborhood conditions affect children’s development and showed that children in deprived neighborhoods were at increased risk for emotional and behavioral problems. Interestingly, while controlling for other factors within the multivariate generalized linear model, the level of neighborhood deprivation showed no effects on the HRQoL and symptoms of anxiety in children and adolescents in Hamburg. This is contrary to findings from Visser and colleagues [32], who found robust associations between neighborhood deprivation and well-being. Also, studies focusing on physical neighborhood factors report mainly positive associations with children’s quality of life, physical health and well-being [53,54]. Evidence focusing on the neighborhood deprivation factors and anxiety symptoms found mainly positive associations in adolescents [55,56]. However, the associations between neighborhood deprivation and internalizing problem behavior, including symptoms of anxiety and depression were less distinct compared to associations with externalizing behavior and well-being in a recent systematic review [32]. Moreover, a study by Vine and colleagues [57] came to mixed results, depending on different types of anxiety. Discrepancies between existing research and our findings might also be explained by pandemic-related phenomena, as most existing research covers the pre-pandemic time.

Regarding our second research question, we did not find an association between levels of neighborhood deprivation and averaged changes in mental health problems and health-related quality of life in children and adolescents during the COVID-19 pandemic in Hamburg. This is in line with the results of a large-scale Irish study by Putra and colleagues [58], who found that neighborhood characteristics did not explain longitudinal changes in mental health problems, suggesting that neighborhood-level factors are more exterior determinates of health and thereby may predict less changes compared to individual-level factors.

In contrast to our findings, a large British longitudinal study with younger children aged 5–8 years found largest mental health deteriorations in children living in the most affluent quintile of areas compared to those living in more deprived areas [11]. The importance of ethnicity is emphasized by Badrick and colleagues [12], who found the largest mental health deterioration in most affluent areas for white children, but in most deprived areas for South Asians. However, it should be noted that our findings are not fully comparable to those of Miall and colleagues [11] and Badrick and colleagues [12] as they used different indicators for operationalizing neighborhood deprivation, which has been shown to affect the strength of association [32]. Further, differences to other studies could be explained by the heterogeneity of pandemic-related restrictions, such as lockdowns and school closures. While these affected all young people in Hamburg in the same way, regardless of where they lived, this could be different in other studies that did not focus on a specific city or area.

It needs to be noted, that the majority of children and adolescents in Hamburg live in areas with medium neighborhood status index [35]. However, our findings indicate, that living in more deprived areas affects young people’s mental health in addition to individual level factors.

Furthermore, it is possible that the neighborhood characteristics already had impacted young people’s mental health prior to participation in the present study (e.g., in their early childhood), as families may have lived in a particular area for a longer period of time. In contrast, the period of two years during the pandemic examined in this study is comparatively short.

We found that individual socioeconomic risk factors such as low parental education, female gender and parental depressive symptoms are especially associated with higher rates of internalizing and externalizing mental health problems. While a low (parental) SES is a well-known risk factors for worse mental health in children and adolescents in pre-pandemic [13] and pandemic studies [11,13,21], evidence focusing on individual and neighborhood socioeconomic status is scarce. Multilevel studies established before the pandemic incorporating individual- and neighborhood-level data suggest a moderating effect of neighborhood disadvantage on the association between family SES and adverse (mental health) outcomes [59,60]. These results are supported by recently published findings from a nationwide cohort study from Denmark, which found that children’s individual SES and neighborhood characteristics are associated with mental health problems in adulthood [28]. Further, Mohan and Barlow [61] found that area-level deprivation did not have a direct impact on mental health status among adults during the pandemic when accounting for other factors, such as individual-level SES and health status. These findings emphasize that area-level indicators for SES cannot replace individual SES indicators. However, as mentioned above, area-level SES can provide further contextual information. Therefore, both individual and area-specific SES measures may be used in combination to investigate patterns of health risks.

According to Galster [62], there are different mechanisms to explain the relation between different neighborhood status factors and behavioral and health outcomes, which might be also applicable to the association between neighborhood deprivation and mental health outcomes. These can be grouped into four broad rubrics. First, social-interactive factors including neighborhood social cohesion have been shown to be associated with better mental health outcomes in young people in a recent review [63]. A study by Aminzadeh and colleagues [64] has found these effects to be strongest in young people from lower socioeconomic backgrounds. In fact, a pre-pandemic review found that social processes are more important than neighborhood status factors in influencing depressive symptoms in adults [65]. Second and third, geographical and institutional factors including access to public services [61] as well as fourth, environmental factors, such as green spaces [66] and perception of neighborhood safety [53], have been found to be associated with better mental health and well-being.

Furthermore, possible moderating and mediating factors between neighborhood deprivation and young people’s mental health problems should also be considered. Although a systematic review found that studies investigating these mechanisms are rare, there is evidence that family and peer processes are important factors. For example, family problems may further exacerbate mental health problems [32]. Moreover, findings from Badrick and colleagues [12] indicate moderating mechanisms of ethnicity.

Finally, findings from the second COPSY Hamburg survey in summer 2022 indicate that mental health problems in Hamburg have not improved and the health-related quality of life in children and adolescents in Hamburg is still impaired [22]. These findings differ from those of the nationwide COPSY study from autumn 2022, where improvements in children and adolescents’ mental health and HRQoL compared to the findings from the first year of the pandemic were found. Specifically, the prevalence of depressive and anxiety symptoms is considerably higher in Hamburg compared to nationwide findings [21]. However, it should be noted that data of the second time point of the COPSY Hamburg study was collected three months before the nationwide data collection took place (i.e., shortly after the loosening of restrictions). Children and adolescents had perhaps not yet sufficiently internalized the new everyday life with fewer or no restrictions. Furthermore, Hamburg had longer, and more extensive restrictions compared to other states in Germany. The fact that Hamburg is a densely populated city state and many families live in apartments with limited living and outdoor space could also have contributed to high levels of depressive and anxiety symptoms as evidence has shown associations between urbanity and a higher prevalence of depression and anxiety [67,68].

### Strengths & limitations

This study has a number of strengths. First, is the prospective character of the study. By using data from 2020 and 2022, we were able to compare findings from two very different time points in the COVID-19 pandemic: Following a strict lockdown in 2020 and two years later, when infection rates were low and most restrictions were lifted. Second, the large size and the representativeness of the sample and the use of internationally established instruments for the assessment of children’ and adolescent’s mental health problems and HRQoL. Third, a neighborhood status index was used as an objective, small-scale measure for neighborhood deprivation in addition to parents self-reported education level as a proxy for individual SES. Considering the above-mentioned debate regarding individual and area-level SES, it’s recommended to incorporate both, individual and neighborhood-level SES.

Nevertheless, the findings of this study have to be seen in light of some limitations. One limitation of the study is the selective use of assessment tools in the self or parent reported questionnaire. Specifically, only the proxy version of the SDQ was employed, resulting in missing data. This choice was made to limit the overall questionnaire length for participating children and adolescents, thereby minimizing potential dropout rates. Due to the relatively low percentage of respondents participating in both measurement points it was decided to treat both time points as independent samples instead of omitting most of the data. However, thus we were waiving the potentials of longitudinal analyses like e.g. a higher statistical power. Yet on the other hand the prerequisites of the independent sample statistical methods were also violated by the fact that the samples are not entirely independent. This might lead to an underestimation of variation in the data and thus a slightly higher chance of significant results. Yet this might be cancelled out by the waiver of applying longitudinal statistical models with their aforementioned higher statistical power. Further, only families with a permanent registration certificate in Hamburg were included in the sample selection, and the questionnaire was only available in German. As a result, particularly vulnerable groups, such as people without a permanent residence or with insufficient knowledge of German, were excluded from participation. These groups may have been of particular importance. All findings are not generalizable to other areas, especially not to other countries or more rural areas due to heterogeneity in the pandemic situation and restrictions within and across countries. However, by focusing on families living in an urban metropole in Germany, where both pandemic situation and restriction measures were homogenous across participants, we could reduce potential confounding effects of heterogeneous restriction measures as in nationwide surveys. Lastly, our lack of longitudinal data precludes causal inferences regarding neighborhood deprivation, the COVID-19 pandemic and mental health outcomes. Furthermore, it has to bear in mind that the found changes over time might be influenced by other factors such as those mentioned above as well as ongoing socio-political crises (e.g. climate change, Russia-Ukraine-war).

### Implications

Previous research has emphasized the importance of familiar resources for a good mental health of children and adolescents during the COVID-19 pandemic [21,22]. Our results expanded the relevance of the social and living environment by underlining the importance of the neighborhood for individual and public mental health. Hence, it is important that existing facilities and low-threshold social services are located in the neighborhood of their recipients. In general, the aim is to integrate health promotion into the everyday lives of children and young people. Therefore, institutions, such as day care centres or schools, are important places for health promotion.

Since the beginning of the COVID-19 pandemic and even before, measures have been taken in the city of Hamburg to provide targeted support for children and young people in order to promote and protect their mental health [69]. For example, in a pilot project (2021–2025), health professionals were deployed in elementary school in socially disadvantaged districts to improve the health literacy of primary school children. Important key stakeholders, teaching staff and parents are involved in this process. Additionally, positions for school psychologists have been created in schools to strengthen psychosocial counselling and support for children and young people in a needs-based and targeted manner [70]. Further measures have been established by the public health insurance institutions, such as the program *MindMatters*. The aim of this measure is to promote the mental health of children and young people at school and to develop a good and healthy school environment [71].

However, as mentioned above, other neighborhood-related influencing factors, e.g. access to green space or recreational areas such as parks or sport facilities as well as neighborhood cohesion, might also have an impact on the psychosocial health [63,66]. Particularly in a densely populated city like Hamburg, urban planners and other (policy) players should take this into account and develop concepts in collaboration with different stakeholders from the health care, social care and educational sectors. One example is the *Hamburg Active City Strategy*, which aims to create opportunities for the citizens of Hamburg “[…] to get active, improve their health and lead an active life in their neighbourhood” [72]. Even if such strategies have already been successfully implemented and expanded, there is need for low-threshold services as well as specialized psychosocial care in the social and neighborhood environment of families. Both, prevention and intervention programs are needed at a local level in order to promote young peoples’ resilience, reduce the burden of mental health disorders and prepare them for future crises.

Further studies should incorporate neighborhood-related factors and focus on potential moderating effects between neighborhood and individual-level SES on mental health outcomes in children and adolescents. Moreover, there is need for internationally validated indicators or scales for assessing neighborhood deprivation to improve understanding of neighborhood-related effects on mental health outcomes ad to allow comparability across studies [32,73].

Last but not least, future research is needed to understand the complex long-term consequences of the COVID-19 pandemic and other ongoing crises on various domains of youth mental health. Therefore, continuous monitoring on statewide, but also local level is highly warranted.

## Conclusion

This study provides empirically based evidence for effective strategies to allocate resources appropriately, make informed urban planning choices, and develop interventions that address the unique challenges faced by individuals and communities in promoting children’s and adolescents’ mental health and improving their overall well-being. In addition to behavioral-based prevention programs in schools and other settings, health promotion should also focus on the creation of a health-promoting living environment such as diverse, socially mixed and green neighborhoods.

Our findings highlight the research need on the relation between neighborhood- and individual SES and young people’s mental health.

## Supporting information

S1 Table Bivariate correlations for mental health outcomes and predictors. (DOCX)
