# Supplementary material for: The impact of neighborhood deprivation on mental health and quality of life in children and adolescents during the COVID-19 pandemic: Findings from the COPSY Hamburg study
Source: PLoS One. 2024 Nov 20;19(11):e0313652. doi: 10.1371/journal.pone.0313652 (PMC11578501; doi:10.1371/journal.pone.0313652)
Supplement: S1 Table — (DOCX) [file pone.0313652.s001.docx]

**Supporting Information**

**S1 Table.** Bivariate correlations for mental health outcomes and predictors.

|  | HRQoL (KIDSCREEN-10) | Internal. symptoms (SDQ) | External. symptoms (SDQ) | Depressive symptoms (CES-DC) | Anxiety symptoms (SCARED) |
| --- | --- | --- | --- | --- | --- |
| Age | **-0.217**** | -0.014 | **-0.179**** | **0.221**** | **0.294**** |
| Female gender | **-0.175**** | -0.037 | **0.184**** | **-0.147**** | **-0.282**** |
| Low parental education^1^ | 0.17 | **-0.076**** | **-0.106**** | -0.042 | 0.004 |
| Medium parental education^1^ | **0.068*** | **-0.096**** | **-0.082**** | **-0.071*** | **-0.104**** |
| Parental depressive symptoms^2^ | **-0.142**** | **0.324**** | **0.232**** | **0.155**** | **0.086**** |
| Neighborhood status index^3^ | **0.066**** | **-0.153**** | **-0.145**** | **-0.089**** | **-0.071**** |
| Time^4^ | **-0.077**** | **0.130**** | 0.025 | **0.071**** | **0.161**** |
| *Notes:* Significant Pearson’s correlations (two-sided) are indicated in bold **p*<0.01, ***p*<0.001; ^1^ according to CASMIN classification; ^2^ according to PHQ-8; ^3^ assessed by RISE; ^4^ participation in COPSY Hamburg T1 (2020) or T2 (2022) | | | | | |
